# Prevalence of Beta-Lactam and Quinolone/Fluoroquinolone Resistance in *Enterobacteriaceae* From Dogs in France and Spain—Characterization of ESBL/pAmpC Isolates, Genes, and Conjugative Plasmids

**DOI:** 10.3389/fvets.2019.00279

**Published:** 2019-08-30

**Authors:** Véronique Dupouy, Mouni Abdelli, Gabriel Moyano, Nathalie Arpaillange, Delphine Bibbal, Marie-Christine Cadiergues, Diego Lopez-Pulin, Sakina Sayah-Jeanne, Jean de Gunzburg, Nathalie Saint-Lu, Bruno Gonzalez-Zorn, Antoine Andremont, Alain Bousquet-Mélou

**Affiliations:** ^1^InTheRes, Université de Toulouse, INRA, ENVT, Toulouse, France; ^2^Da Volterra, Paris, France; ^3^Departamento de Sanidad Animal, Facultad de Veterinaria y Centro de Vigilancia Sanitaria Veterinaria (VISAVET), Universidad Complutense de Madrid, Madrid, Spain; ^4^UDEAR, Université de Toulouse, INSERM, ENVT, Toulouse, France; ^5^Centro Veterinario Loranca, Fuenlabrada, Spain

**Keywords:** dog, feces, antibiotic resistance, ESBL/pAmpC, fluoroquinolone, plasmid

## Abstract

Quantitative data on fecal shedding of antimicrobial-resistant bacteria are crucial to assess the risk of transmission from dogs to humans. Our first objective was to investigate the prevalence of quinolone/fluoroquinolone-resistant and beta-lactam-resistant *Enterobacteriaceae* in dogs in France and Spain. Due to the particular concern about possible transmission of extended-spectrum cephalosporin (ESC)-resistant isolates from dogs to their owners, we characterized the ESBL/pAmpC producers collected from dogs. Rectal swabs from 188 dogs, without signs of diarrhea and that had not received antimicrobials for 4 weeks before the study, were quantified for total and resistant *Enterobacteriaceae* on selective media alone or containing relevant antibiotic concentrations. Information that might explain antibiotic resistance was collected for each dog. Extended-spectrum cephalosporin-resistant isolates were subjected to bacterial species identification (API20E), genetic lineage characterization (MLST), ESBL/pAmpC genes identification (sequencing), and plasmid characterization (pMLST). Regarding beta-lactam resistance, amoxicillin- (AMX) and cefotaxime- (CTX) resistant *Enterobacteriaceae* were detected in 70 and 18% of the dogs, respectively, whereas for quinolone/fluoroquinolone-resistance, Nalidixic acid- (NAL) and ciprofloxacin- (CIP) resistant *Enterobacteriaceae* were detected in 36 and 18% of the dogs, respectively. Medical rather than preventive consultation was a risk marker for the presence of NAL and CIP resistance. CTX resistance was mainly due to a combination of specific ESBL/pAmpC genes and particular conjugative plasmids already identified in human patients: *bla*_CTX−M−1_/IncI1/ST3 (*n* = 4), *bla*_CMY−2_/IncI1/ST12 (*n* = 2), and *bla*_CTX−M−15_/IncI1/ST31 (*n* = 1). *bla*_SHV−12_ (*n* = 3) was detected in various plasmid lineages (InI1/ST3, IncI1/ST26, and IncFII). ESBL/pAmpC plasmids were located in different genetic lineages of *E. coli*, with the exception of two strains in France (ST6998) and two in Spain (ST602). Our study highlights dogs as a potential source of Q/FQ-resistant and ESBL/pAmpC-producing bacteria that might further disseminate to humans, and notably a serious risk of future acquisition of CTX-M-1 and CMY-2 plasmids by the owners of dogs.

## Introduction

Extended-spectrum and plasmidic-AmpC beta-lactamases (ESBL/pAmpC), which cause resistance to extended-spectrum cephalosporins (ESCs), are of considerable concern in veterinary and human medicine. This is because resistance to ESCs and co-resistance to other antimicrobial families (e.g., fluoroquinolones) limits the treatment options for infections with ESBL/pAmpC-producing bacteria. ESCs and fluoroquinolones, also used to treat animals, have been identified as critically important antibiotics in human medicine (1).

The emergence of ESBL/pAmpC-producing bacteria in animals and the environment is raising serious concerns (2, 3). In particular, the gastrointestinal tract of animals, including domestic pets, may serve as reservoirs of resistant bacteria that can cause opportunistic infections in vulnerable dogs and could be a source of contamination for humans. For example, dogs could possibly transmit ESC-resistant bacteria due to their close contacts with humans, the high consumption of beta-lactams in small animal veterinary practice (4), and the frequent occurrence of ESBL/pAmpC-producing *Escherichia coli* (5). Recently, human-related pathogenic strains have been identified in dogs (6), and the sharing of identical ESBL/pAmpC strains between humans and dogs from the same households has also been demonstrated (7).

Although ESBL/pAmpC producers can spread clonally (8), there is evidence that mobile genetic elements carrying antimicrobial resistant genes can be transferred between bacteria, notably from commensal to pathogenic *Enterobacteriaceae* (9). Many studies have highlighted the existence of different *E. coli* bacteria harboring *bla*_CTX−M_ or *bla*_CMY−2_-carrying plasmids, some being shared between human and animal strains (5). Such successful plasmids efficiently contribute to the spread of resistance determinants (10). In addition, the fact that many strains harboring *bla*_CTX−M−1_ or *bla*_CMY−2_ genes are resistant to other antimicrobial classes may also increase the threat. The use of expanded-spectrum cephalosporins and of fluoroquinolones in dogs is known to select ESBL/pAmpC producers in the fecal microbiota (11–13).

Over the past 5 years, the presence of ESBL/pAmpC genes in *Enterobacteriaceae* strains from the feces of healthy dogs in Europe has been reported in several studies (5, 7, 14–16). However, information on the clonality of the ESBL/pAmpC isolates and knowledge about the plasmids carrying these ESBL/pAmpC genes is much more limited (5, 15, 17, 18).

Thus, examination of the occurrence of ESBL/pAmpC bacteria together with plasmids circulating in healthy pets, and their associated co-resistance, is of major importance with respect to the risk of transfer to humans in daily situations of close contact and will also extend understanding of the dissemination and persistence of ESBL and pAmpC beta-lactamase genes.

The present study was designed to assess the prevalence of beta-lactam and quinolone/fluoroquinolone resistance in fecal *Enterobacteriaceae* from dogs in Spain (Madrid) and France (Toulouse), to identify the potential risk factors for the presence of antibiotic resistance, and to characterize the ESC-resistant isolates, the genes coding for and plasmids carrying ESBL/pAmpC resistance in those isolates.

## Materials and Methods

### Isolation of *Enterobacteriaceae* Strains

Between March 2013 and January 2014, fecal specimens from 269 different dogs presented at two veterinary clinics (158 at the small animal clinics of the veterinary teaching hospital in Toulouse, France and 111 at a veterinary clinic in Madrid, Spain) were sampled. These dogs were being admitted for a preventive health service, vaccination or medical consultation (disease, trauma, etc.). Dogs were included in the study if they had not received any systemic or local antimicrobial during the previous 4 weeks, according to the owner. Animals with diarrhea were not included in the study. Breed, age, weight, diet (commercial, homemade, other), and reason for the visit (preventive, medical) were recorded for each dog. According to EU law (Directive 2010/63/UE), procedures which use animals according to common veterinary practice did not require minister permission and ethics committee opinion. Sampling were performed by veterinarians in veterinary clinics. They consisted in non-invasive samples (rectal swabbing) performed on client-owned animals and were acquired with the written consent from all owners.

Samples were collected by swabbing, immediately suspended in liquid amies preservation medium (Copan eSwabs Amies liquid, Biomerieux), stored at +4°C and analyzed within 24 h after sampling. Ten-fold dilutions were prepared in 0.9% sterile saline. One hundred microliter were inoculated (i) on MacConkey agar not supplemented with antimicrobials to obtain counts of total *Enterobactericeae*; (ii) on MacConkey agar supplemented with amoxicillin (AMX 100 μg/mL) or cefotaxime (CTX 1.5 μg/mL) to obtain counts of penicillin- or cefotaxime-resistant *Enterobacteriaceae*, and (iii) on MacConkey agar supplemented with nalidixic acid (NAL 20 μg/mL) or ciprofloxacin (CIP 2 μg/mL) to obtain counts of quinolones- (Q) or fluoroquinolones- (FQ) resistant *Enterobacteriaceae*. These antibiotic concentrations have been chosen according to the European Committee on Antimicrobial Susceptibility Testing Clinical breakpoint (www.eucast.org) to obtain non-susceptible/resistant *Enterobacteriaceae*.

All batches of media supplemented with antibiotics were tested with positive (resistant) and negative (susceptible) control strains. Total or resistant *Enterobacteriaceae* (cfu/mL) were counted after incubating these MacConkey agar plates for 24 h at 37°C. The limit of detection was 10 cfu/mL of undiluted liquid preservative medium. The prevalence of dogs carrying resistant *Enterobacteriaceae* (%) was estimated. Samples containing at least 10^4^ total *Enterobacteriaceae*/mL were included in the analysis to limit the risk of underestimating the prevalence. Of the 269 fecal swabs collected, 188 (*n* = 90 from Toulouse and *n* = 98 from Madrid) were included in the analysis.

Multivariate logistic regression analyses were performed to identify independent predictors (sampling site, age, weight, diet, and reason for visit) of resistance carriage (prevalence of AMX-R, CTX-R, NAL-R, and CIP-R) among the investigated animals, using Systat v13.1 version. Statistical significance was set at *p* < 0.05. Comparisons of resistance abundance were done using a chi-square test with the significance level set at 0.05.

### Identification and Typing of CTX-Resistant Bacteria

CTX-resistant colonies of *Enterobacteriaceae* were collected (one colony per animal), and identified by using API 20E galleries (bioMérieux). Non-*Enterobacteriaceae* isolates (mostly *Pseudomonas* spp.) were discarded.

Multilocus sequence typing (MLST) was carried out according to the protocol described on the *E. coli* (http://mlst.warwick.ac.uk/mlst/dbs/Ecoli) and *C. freundii* (https://pubmlst.org/cfreundii) MLST website.

### Antibiotic Susceptibility Testing

The antibiotic susceptibilities of CTX-R *E. coli* and their transconjugants were determined by disk diffusion method according to CLSI protocols (19). The following antibiotic disks were used: ampicillin (10 μg), amoxicillin (20 μg) plus clavulanic acid (10 μg), cefoxitin (30 μg), ceftazidime (30 μg), cefotaxime (30 μg), cefepime (30 μg), aztreonam (30 μg), imipenem (10 μg), streptomycin (10 μg), gentamicin (10 μg), kanamycin (30 μg), nalidixic acid (30 μg), ciprofloxacin (5 μg), tetracycline (30 μg), chloramphenicol (30 μg), trimethoprim (5 μg), and sulfonamides (300 μg) (Bio-Rad, Marnes-la-Coquette, France if available or Oxoid, Dardilly, France). The susceptibility breakpoints for all antimicrobials were those recommended by CLSI (20).

ESBL production was detected by double-disk synergy test on Mueller-Hinton agar between clavulanic acid and ceftazidime, cefotaxime, or cefepime (20, 21). AmpC beta-lactamases detection was based on the inhibitory effect of cloxacillin on AmpC production observed on plates supplemented with 200 mg/L cloxacillin. The control strains used were *E. coli* ATCC 25922, *K. pneumoniae* ATCC 700603, and *K. pneumoniae* CMY-2 from Pr R. Bonnet, France.

### Characterization of *bla_*ESBL*/*AmpC*_* Genes

After bacterial DNA extraction with a DNeasy blood and tissue kit (Qiagen, Hilden, Germany), the *bla*_TEM_, *bla*_SHV_, *bla*_CTX−M_, *bla*_CMY−2_, genes were detected by PCR and sequenced using specific previously-described primers (22, 23). Sequences were analyzed by the BLAST Internet services (www.ncbi.nlm.nih.gov/BLAST).

### Transferability of the *bla*ESBL/pAmpC Genes and Plasmid Characterization

Plasmids were transferred by conjugation to rifampin-resistant *E. coli* recipient strains. One-milliliter of a LB culture of the ESBL/AmpC-producing donor strain (10^8^ cfu/mL) and one mL of the J0C0J7-rifR4 rifampicin-resistant recipient (10^8^ cfu/mL) strain were added to 3 mL LB broth and incubated for 2 h at 37°C without agitation. Transconjugants were selected on LB agar containing 1.5 μg/mL of cefotaxime and 100 μg/mL of rifampicin. Plasmid DNA from transconjugants was purified and resolved by electrophoresis in 0.7% agarose, as described previously (24), to confirm that cells carried only one conjugative plasmid.

Plasmid replicon types were determined using the PCR-based replicon typing (PBRT) scheme (25) and plasmid sequence subtypes by applying plasmid multilocus sequence typing (pMLST) for IncI1 (http://pubmlst.org/plasmid/).

## Results

### Prevalence of Resistant *Enterobacteriaceae*

The characteristics of the dog population studied are given in Table 1. A total of 188 dogs (90 from Toulouse and 98 from Madrid), with ages ranging from 4 months to 16 years (median age 5 years), and feces containing at least 10^4^ cfu/mL of total *Enterobacteriaceae*, were included in the study. Body weights ranged from 1 to 53 kg (median weight 10 kg), and most of the dogs (73%) were fed with commercial feed. Eighty-four dogs (44.5%) were admitted for medical consultation, mainly in Toulouse (36.2%), most of them for skin disorders, whereas 94 dogs (50.0%) were admitted for preventive consultation, mainly in Madrid (38.8%), the majority being admitted for vaccination.

**Table 1:** Distribution of the predictor variable from dogs in Madrid and Toulouse veterinary clinics.

**Risk marker**		**Madrid**	**Toulouse**	**Total**
		***N* (%)**	***N* (%)**	***N* (%)**
Reason for consultation	Medical	16 (9)	68 (36)	84 (45)
	Preventive	73 (39)	21 (11)	94 (50)
	Unknown	9 (5)	1 (1)	10 (5)
Diet	Commercial	76 (40)	61 (32)	137 (73)
	Home	4 (2)	4 (2)	8 (4)
	Mixed/other	10 (5)	6 (3)	16 (9)
	Unknown	8 (4)	19 (10)	27 (14)
Age (year)	≤ 5	54 (29)	48 (26)	102 (54)
	>5	42 (22)	39 (21)	81 (43)
	Unknown	2 (1)	3 (2)	5 (3)
Sex	Female		50 (27)	
	Male		39 (21)	
	Unknown	98 (52)	1 (1)	
Weight (Kg)	>20	17 (9)	37 (20)	54 (29)
	>10–20	13 (7)	16 (9)	29 (15)
	≤ 10	66 (35)	33 (18)	99 (53)
	Unknown	2 (1)	4 (2)	6 (3)

**Figure 1:**
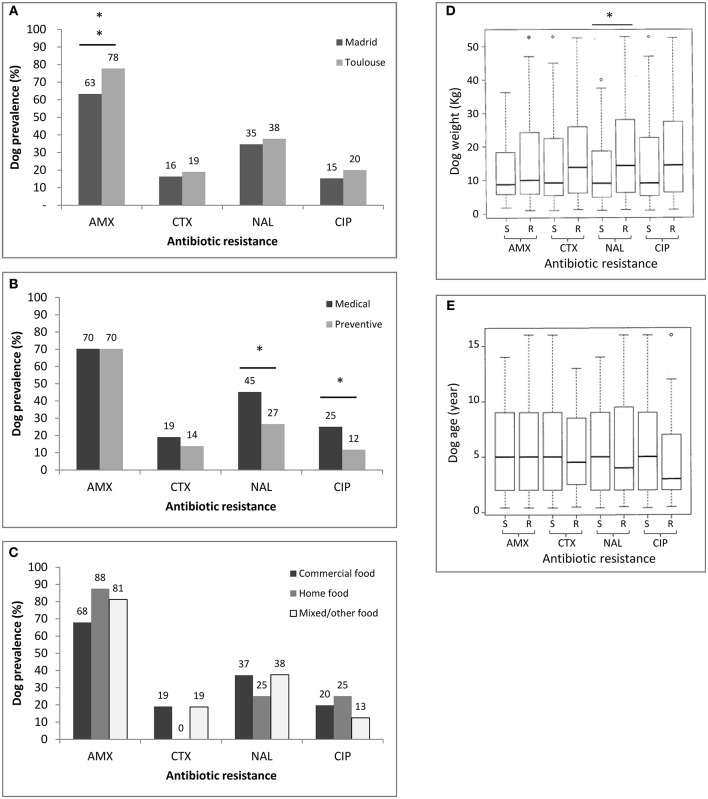
Prevalence of *Enterobacteriaceae* resistant to amoxicillin (AMX), cefotaxime (CTX), nalidixic acid (NAL), and ciprofloxacin (CIP) in the population of 188 dogs without recent antimicrobial treatment according to the predictor variable. Antibiotic resistance according to site of sampling (Madrid vs. Toulouse) **(A)**, the reason for consultation (Medical vs. preventive) **(B)**, the type of diet **(C)**, dog weight **(D)**, and dog age **(E)**. Possible associations between sampling site, age, weight, diet, reason for visit, and carriage of antibiotic resistance were analyzed by logistic regression models. Asterisks indicate a significant difference, **p < 0.01 and *p < 0.05. S, susceptible; R, resistant.

The prevalences of dogs carrying beta-lactam- or Q/FQ-resistant bacteria and its association with predictor (explanatory) variables are shown in Table 2 and Figure 1. Regarding the beta-lactam-resistant *Enterobacteriaceae* in feces, approximately 70% of the dogs carried AMX-R and 18% carried CTX-R *Enterobacteriaceae* (19% in Toulouse and 16% in Madrid). Multivariate regression analysis revealed that dogs in Toulouse were more frequently colonized by AMX-R *Enterobacteriaceae* (78%) than those from Madrid (63%; *p* < 0.01). The reason for consultation, diet, age and weight were not predictors of beta-lactam resistance carriage among animals (Table 2 and Figure 1).

**Table 2:** Multivariate logistic regression analysis for predictor variable associated with antibiotic resistance carriage.

**Predictor variable**			**Amoxicillin resistance**				**Cefotaxime resistance**				**Nalidixic acid resistance**				**Ciprofloxacin resistance**		
		**OR**	**95% CI**		***p*-value**	**OR**	**95% CI**		***p*-value**	**OR**	**95% CI**		***p*-value**	**OR**	**95% CI**		***p-*value**
	**Dogs**		**lower**	**upper**			**lower**	**upper**			**lower**	**upper**			**lower**	**upper**	
**Site**																	
Toulouse	90																
Madrid	98	3.635	1.375	9.606	** <0.01**	1.112	0.379	3.257	0.847	0.507	0.206	1.250	0.140	0.598	0.206	1.735	0.344
**Reason for consultation**																	
Medical	84																
Predictive	94	0.646	0.262	1.592	0.343	1.119	0.401	3.121	0.831	3.013	1.289	7.046	** <0.05**	3.370	1.205	9.425	** <0.05**
**Diet**																	
Commercial	137																
Home	8	0.395	0.101	1.547	0.182	0.759	0.191	3.013	0.695	0.682	0.213	2.181	0.519	1.634	0.328	8.132	0.549
Mixed/other	16	1.315	0.109	15.865	0.829	0.000	0.000	-	0.999	0.660	0.094	4.636	0.676	2.996	0.315	28.459	0.339
**Age**	183	0.973	0.891	1.062	0.536	0.944	0.842	1.058	0.324	0.987	0.904	1.077	0.768	0.950	0.855	1.056	0.345
**Weight**	182	1.009	0.974	1.046	0.615	1.022	0.983	1.062	0.283	1.048	1.013	1.084	** <0.01**	1.021	0.984	1.060	0.265

*Multivariate logistic regression analyses were performed to identify independent predictors (sampling site, age, weight, diet, and reason for visit) of antibiotic resistance carriage among the investigated animals. Statistical significance was set at p < 0.05 and is indicated in bold*.

Regarding quinolone/fluoroquinolone-resistant *Enterobacteriaceae*, the overall frequency of carriage of NAL-R bacteria was 36% (38% in Toulouse and 35% in Madrid) and that of CIP-R bacteria was 18% (20% in Toulouse and 15% in Madrid). The multivariate logistic regression analysis indicated that the reason for the dog's visit to the veterinary clinic was a predictor of Q/FQ resistance (*p* < 0.05). Animals brought in for preventive measures were less likely to carry *Enterobacteriaceae* resistant to Q/FQ than those presented for medical treatment (27 vs. 45% for NAL and 12 vs. 25% for CIP). Dog weight was also found to be a predictor of resistance prevalence, but only for NAL (*p* < 0.01). The population of dogs harboring fecal bacteria resistant to NAL weighed more (median = 14.30 kg) than the population of dogs without resistant bacteria (median = 9.10 kg). A similar trend, but without statistical significance, was apparent for other antibiotics (CTX and CIP).

### Characterization of ESBL/AmpC Producers, Genes, Plasmids

ESBL/AmpC genes and the plasmids carrying them were identified in 14 cefotaxime-R *Enterobacteriaceae* isolated from the feces (Table 3). They included 10 ESBL-producing *E. coli* and 4 AmpC-carrying strains (2 *E. coli*, 1 *P. mirabilis* having acquired AmpC enzyme *and* 1 *C. freundii that carries naturally a chromosomal AmpC*). According to the MLST database, the 12 *E. coli* were associated with 10 different sequence types (STs) of which nine have been previously reported. ESBL-producing *E. coli* presented multiple associated resistances. All strains were resistant to sulfonamides and tetracycline, and some to trimethoprim (5/10), chloramphenicol (4/10), and streptomycin (2/10). ESBL-producers in Madrid were always co-resistant to NAL and CIP whereas those isolated in Toulouse were not, despite the presence of a significant level of Q/FQ resistant strains in the feces. The ESBL phenotype was mostly due to the *bla*_CTX−M−1_ gene (*n* = *6*), whereas the *bla*_CTX−M−15_ gene (*n* = 1 in Toulouse) was rare. The 6 *bla*_CTX−M−1_ genes were systematically carried by the conjugative IncI1/ST3 plasmid subtype that conferred additional resistance to tetracyclines and sulphonamide. The *bla*_CTX−M−1_ IncI1/ST3 plasmid was found in isolates belonging to four different STs (ST6998 (*n* = 2), ST227 (*n* = 1), and ST38 (*n* = 1) in Toulouse and ST602 (*n* = 2) in Madrid). The *bla*_CTX−M−15_ gene was on a conjugative IncI1/ST31 plasmid which did not confer other resistances to its host *strain* which belonged to ST2449. Three *bla*_SHV−12_ genes were also identified, only in Madrid, and were carried by a diversity of conjugative plasmids found in MLST clonally unrelated *E. coli*: *bla*_SHV−12_ IncI1/ST3 plasmid associated with ST453(STC86), *bla*_SHV−12_ IncI1/ST26 with a new ST and *bla*_SHV−12_ IncFII plasmid with ST1594.

**Table 3:** Extended-spectrum cephalosporinase of *Enterobacteriaceae* isolated from feces of healthy dogs in France and Spain and molecular characterization of *bla*_CTX−M_ and *bla*_CMY_ genes and the plasmids carrying them.

**Strain**	**Site**	**Species**	**Sequence type (ST) ST complex (STC)**	**Phenotype**	**Non-beta-lactam resistance Pattern**	**Phenotype transferred**	***bla* gene transferred**	**Co-transferred non-beta-lactam resistance**	**Plasmid replicon type**	**Plasmid sequence type**
T22	Toulouse	*E. coli*	ST227 (STC10)	ESBL	SSS TET	ESBL	*bla*_CTX−M−1_	SSS TET	IncI1	ST3
T39	Toulouse	*E. coli*	ST38 (STC38)	ESBL	SSS TMP TET	ESBL	*bla*_CTX−M−1_	SSS TET	IncI1	ST3
T65	Toulouse	*E. coli*	ST2449	ESBL	SSS TMP TET	ESBL	*bla*_CTX−M−15_	None	IncI1	ST31
T80	Toulouse	*C. freundii*	New ST	AmpC^*^	TET KAN STR CHL					
T115	Toulouse	*E. coli*	ST6998	ESBL	SSS TET	ESBL	*bla*_CTX−M−1_	SSS TET	IncI1	ST3
T116	Toulouse	*E. coli*	ST6998	ESBL	SSS TET	ESBL	*bla*_CTX−M−1_	SSS TET	IncI1	ST3
M9	Madrid	*E. coli*	ST453 (STC86)	ESBL	SSS TMP TET STR CIP NAL	ESBL	*bla*_SHV−12_	None	IncI1	ST3
M40	Madrid	*E. coli*	New ST	ESBL	TET CIP NAL	ESBL	*bla*_SHV−12_	TET	IncI1	ST26
M49	Madrid	*E. coli*	ST1594	ESBL	SSS TET STR CHL CIP NAL	ESBL	*bla*_SHV−12_	SSS CHL	IncFII	76
M53	Madrid	*E. coli*	ST12 (STC12)	AmpC	SSS TET STR CHL CIP NAL	AmpC	*bla*_CMY−2_	SSS TET STR CHL	IncI1	ST12
M76	Madrid	*E. coli*	ST602 (STC446)	ESBL	SSS TMP TET CHL CIP NAL	ESBL	*bla*_CTX−M−1_	SSS TET	IncI1	ST3
M104	Madrid	*E. coli*	ST602 (STC446)	ESBL	SSS TMP TET CHL CIP NAL	ESBL	*bla*_CTX−M−1_	SSS TET	IncI1	ST3
M107	Madrid	*P. mirabilis*	Not applicable	AmpC^*^	TET NAL					
M110	Madrid	*E. coli*	ST10 (STC10)	AmpC	STR	AmpC	*bla*_CMY−2_	STR	IncI1	ST12

*SSS, sulfonamides; TMP, trimethoprim; TET, tetracycline; CHL, chloramphenicol; KAN, kanamycin; STR, streptomycin; CIP, ciprofloxacin; NAL, nalidixic acid*.

* *strain amplified the bla_CMY_ gene*.

AmpC beta-lactamases were detected in 4 isolates: 1 *C. freundii* isolated from Toulouse dogs and 2 *E. coli*, 1 *P. mirabilis* from Madrid. As CMY-2 is the most common type of AmpC and pAmpC enzymes among *Enterobacteriaceae*, we have focused on the detection of *bla*_*CMY*−2_ *gene*. For the 2 *E. coli*, belonging to ST10 and ST12, the *bla*_CMY−2_ gene was carried by the IncI1/ST12 conjugative plasmid, one carrying only streptomycin resistance while the second was associated with multiple resistances such as sulfonamide, tetracycline, streptomycin and chloramphenicol. The remaining 2, the *bla*_CMY−2_ gene in *P. mirabilis* and the *bla*_CMY−80_ gene in *C. freundii*, were not located on conjugative plasmids.

## Discussion

The resistance phenotypes investigated in this study are of high clinical relevance in small animal veterinary practice in view of the widespread use of beta-lactams and fluoroquinolones for treating common infections in dogs (4) and the potential risk of resistance transmission (bacteria or genes) to humans through direct contact (1).

### Prevalence of Resistant *Enterobacteriaceae*

Even if comparison between different studies is difficult due to biases associated with methodological factors, the high prevalences of AMX-R (70%) and CTX-R (18%) *Enterobacteriaceae* were similar to those already reported, over the last 5 years, in healthy dogs in various European countries (5, 14, 26, 27), but higher than those reported in Denmark, Sweden and the UK (7, 15, 28). In our study, the prevalence of AMX-R isolates included *Enterobacteriaceae* species naturally resistant to penicillin like *K. pneumoniae, C. freundii, Enterobacter* and those having acquired such resistance like *E. coli, P. mirabilis*.

Similarly, a high prevalence of NAL-R (35–38%) and CIP-R (15–20%) carriers was detected, as previously reported in Portugal (27), but higher than that reported in the UK (29). The use of critically important antimicrobials (3rd and 4th generation cephalosporin and quinolone/fluoroquinolone) is more commonly cited as being prescribed for dogs in Spain, France and Germany than in Sweden and the UK (4). We therefore believe that the observed high prevalence of cefotaxime (CTX) and Q/FQ resistance in dogs may be associated with the use of critically important antimicrobials for canine treatments.

The prevalence of AMX-R was higher in Toulouse (78%) than in Madrid (63%) but such result is difficult to interpret in light of antibiotic usage data described in the literature for the two countries (4). Dog weight (Kg) was a risk marker significantly associated with nalidixic acid resistance whereas age was not. This result is difficult to explain but the possible difficulty of precisely administering the recommended dose according to the dog's weight might sometimes lead to over-dosing (over-exposure of fecal flora to antibiotics) or under-dosing (which might necessitate a change of antibiotherapy), both situations promoting the development of antibiotic resistance.

The significantly higher prevalence of NAL-R and CIP-R in dogs presented for medical consultation has already been reported (27) and might be related to the fact that healthy animals have fewer opportunities for contact with antimicrobials. However, the past medical histories of these dogs were not recorded. Exposure to quinolones had previously been indicated as a risk factor for the emergence of resistance to several antibiotics in *E. coli* isolated from dogs (27) but the involvement of other antimicrobial classes (e.g., tetracyclines, sulfonamides, aminoglycosides), that are frequently used in canine therapy, cannot be excluded. Because no antimicrobial was administered to the dogs during the month prior to sampling, our results are consistent with the hypothesis that the reversibility of resistance in the absence of antimicrobials is a slow process.

It is noteworthy that as almost 20% of the dogs, independently of origin, were CTX-R (ESBL or AmpC) or FQ-R carriers, the owners (including family members) could be at risk of acquiring these resistant bacteria and genes from their dogs (7) especially if they are carried by conjugative plasmids. FQ resistance was mainly mediated by mutation in chromosomic genes whereas plasmid-mediated FQ resistance (PMQR) determinants only confer low-level resistance to fluoroquinolones. On the 16 CIP-R and 27 NAL-R *Enterobacteriaceae* isolated in our study, we performed the detection for predominant PMQR *qnrA, qnrB, qnrS*, and *aac(6*′*)- Ib-cr* by PCR as described elsewhere (30, 31). No PMQR were detected (data not shown) but we know that among fluoroquinolone non-wild *Enterobacteriaceae*, only 20% carried at least one PMQR determinant (32).

### Characterization of ESBL/AmpC Producers, Genes, and Plasmids

Among the ESBL genes identified in *Enterobacteriaceae* isolated from different dogs in the two countries, the *bla*_CTX−M−1_ gene was exclusively located on the specific conjugative IncI1/ST3 plasmid. Although this study is the first to identify *bla*_CTX−M−1_ on IncI1/ST3 plasmid in Spain, such a combination has recently been reported in France in healthy urban dogs (5), in both diseased and healthy humans (33) but also in food-producing animals and the environment (22, 34). The *bla*_CTX−M−1_ on IncI1/ST3 plasmid was detected in four different *E. coli* ST, including ST38 and ST602 which have frequently been identified in different situations (human pathogens or not, livestock, environment) but are only rarely found in dogs. The *E. coli* ST38 and ST602 lineages carrying *bla*_CTX−M−1_ on IncI1/ST3 plasmids have previously been reported in food-producing animals in Portugal (35) and healthy dogs (36) in Tunisia, respectively. The diversity of the analyzed strains (date and place of isolation) and the diversity of the *E. coli* ST, in contrast to the homogeneity of the *bla*_CTX−M−1_ plasmid type, confirm the specific capability of the *bla*_CTX−M−1_-carrying IncI1/ST3 plasmid to disseminate through unrelated bacterial strains.

In our study, one *bla*_CTX−M−15_/IncI1/ST31 conjugative plasmid, carrying an additional *bla*_TEM−1_ gene without additional resistance, was identified in a French isolate. Such a plasmid has already been detected in *E.coli* and *Shigella sonnei* clinical isolates from cattle and humans in different European countries (37, 38). This plasmid was found in *E. coli* ST2449 which has been reported once for a human pathogen in Europe and twice on the American continent for animal pathogens (http://enterobase.warwick.ac.uk/species/ecoli/search_strains).

Three *bla*_SHV−12_ genes were identified only in Spain and were carried by three different conjugative plasmids: the *bla*_SHV−12_ IncI1/ST3 plasmid previously reported in 2 *E. coli* of poultry origin in Italy (39), the *bla*_SHV−12_ IncI1/ST26 plasmid previously identified in *E. coli* of human origin in Italy (39), from food animals and products in Portugal (35) and the *bla*_SHV−12_ F76:A-:B- FII plasmid not yet described. In addition, SHV-12-carrying plasmids were located in three different genetic lineage strains. Among them, a new ST and 2 other STs (ST453, ST1594) that have occasionally been detected in livestock and poultry on different continents as well as in the environment and humans for ST453 (40, 41). The ability of *bla*_SHV−12_ genes to combine with different plasmids allows the enzymes to attain diverse niches worldwide, as previously demonstrated (42). Prevalence of the *bla*_SHV−12_ gene seemed higher in Spain but the number of cefotaxime-R isolates in France was too small to draw a conclusion.

Three AmpC were detected among the eight cefotaxime-R *Enterobacteriaceae* in Spain but only one in France. The *bla*_CMY−2_ on IncI1/ST12 plasmid was the only conjugative plasmid-encoded p*Amp*C gene that we detected in two different *E. coli* lineages (ST10 and ST12). The *E. coli* ST10 and ST12 have repeatedly been isolated from various reservoirs i.e., healthy or diseased animals including dogs, humans and the environment (40). Previous studies support the hypothesis that plasmids play the major role in the transmission of *bla*_CMY−2_ between reservoirs. The widespread distribution of *bla*_CMY−2_-carrying IncI1/ST12 plasmid has been reported among various *E. coli* STs from human patients, livestock animals, food, environment, and also from healthy dogs in Tunisia (17, 35, 36, 41, 43). However, to our knowledge, this is the first time that the *bla*_CMY−2_ IncI1/ST12 plasmid has been identified in healthy dogs in Europe although it was recently identified in a single clinical isolate associated with another ST in Denmark (17). Until now, the *bla*_CMY−2_ on IncI1/ST2 plasmid was the only one reported in healthy dogs in Europe (5, 11, 17). *E. coli* ST10 harboring *bla*_CMY−2_ on the IncI1/ST12 plasmid seems to circulate in European broiler production (17). In addition to food animals, which are often proposed as possible reservoirs for *bla*_CMY−2_ in humans through the spread of specific plasmids, our results also raise questions about the possible contribution, to this burden in Europe, of *bla*_CMY−2_-carrying IncI1/ST12 plasmid from companion animals.

The third AmpC-carrying isolate in Spain was a CMY-2-producing *Proteus mirabilis* in which mobilization of the *bla*_CMY−2_ gene does not involve a plasmid. Hybridization experiments are needed to confirm the chromosomal location of this *bla*_CMY−2_ gene. In France, a single CMY-80-producing *Citrobacter freundii* was found in canine feces and the bla_CMY_ variant gene was not carried by a conjugative plasmid. Such a *bla*_CMY_ variant was only found in *C. freundii* isolated from human urine samples in Spain (GenBank Accession Number JQ733577).

Overall, a diversity of *bla*_ESBL/pAmpC_ genes were found but the *bla*_CTX−M−15_ which is highly predominant in the ESBL-producers in human and dog pathogens seems minor in the commensal digestive flora (44–47). In the other hand, *bla*_CTX−M−1_ and *bla*_SHV−12_ have recently emerged in dog pathogens (45). ESBL/AmpC genes were found in different clonal lineages of *E. coli* but not in the most prevalent ST in human (ST131, ST648) and dog (ST410) pathogens. However, some successful ESBL/pAmpC plasmid lineages have been described in human and dog pathogens.

In our study, most of the ESBL/AmpC strain isolated from Madrid presented the broadest multiresistance pattern, showing co-resistance to ESC and FQ. Similarly, such co-resistance in *E. coli* from food or food-producing animals in Europe was shown to occur at the highest frequency in Spain (48). We performed the detection for predominant PMQR *qnrA, qnrB, qnrS*, and *aac(6')- Ib-cr* by PCR as described elsewhere on ESBL/AmpC strain but no PMQR were detected except a qnrA gene in the AmpC-producer C. freundii (data not shown). In our study, the low prevalence of PMQR contrast with the high prevalence previously described in dog pathogens (32, 44, 49). Anyway, multiresistance to different classes of antibiotic used in veterinary medicine, conferred by conjugative plasmids and/or by the host strains, may increase the potential risk of co-selection, maintenance, transmission, and propagation of the multidrug-resistant *E. coli* isolates and/or multidrug-resistant plasmids in digestive tract. Moreover, despite the few strains studied here, and in addition to the greater multiresistance reported in Spain, our results suggest that the diversity of ESBL/pAmpC genes and plasmids is greater in Spain than in France. This may be due to a geographical factor, differences in the use of antibiotics in dogs or differences in the distribution of ESBL/Ampc strains and plasmids in the environment of dogs (humans, water, and food). The sharing of ESBL/pAmpC-carrying plasmids between healthy dogs and humans is of particular concern.

In conclusion, our study reveals a high prevalence of resistance to fluoroquinolones (18%) and extended-spectrum-cephalosporins (18%) in feces from dogs in Toulouse and Madrid. The state of health of the animals is a risk factor for quinolone-resistance carriage, probably related to previous treatments. In unrelated dogs in Spain and/or France, the spread of extended-spectrum-cephalosporinases can be due to successful combination of particular ESBL/pAmpC genes and specific plasmid ST, as for *bla*_CTX−M−1_/IncI1/ST3 and *bla*_CMY−2_/IncI1/ST12. On the contrary, the *bla*_SHV−12_ gene combines with different plasmid lineages. Such highly conjugative plasmids have previously been described in healthy or pathogenic *E. coli* isolates of human, animal and food origin. They are located in different *E. coli* ST, and some are increasingly being identified in various known reservoirs. Considering the prevalence of resistance to fluoroquinolones and extended-spectrum-cephalosporinases (notably the large reservoir of CTX-M-1 and CMY-2 producers in dogs) there is a serious and plausible risk of future acquisition of CTX-M-1 and CMY-2 plasmids by their owners.

## Data Availability

The datasets generated for this study are available on request. The raw data supporting the conclusions of this manuscript will be made available by the authors, without undue reservation, to any qualified researcher.

## Author Contributions

VD, MA, SS-J, NS-L, BG-Z, AA, and AB-M designed the study. M-CC and DL-P collected the feces. VD, MA, GM, DB, and NA performed bacterial counts and the storage of resistant isolates. VD carried out the statistical tests and analyzed the DNA sequences. VD and NA analyzed the ESBL/AmpC isolates. VD, JdG, BG-Z, AA, and AB-M drafted the manuscript. All the authors edited the manuscript and approved the paper.

### Conflict of Interest Statement

MA, SS-J, and NS-L are employees of Da Volterra. JdG and AA are consultants of Da Volterra. The authors declare that this study received funding from the SME Da Voltera. The funder had the following involvement with the study: study design, data collection, the writing of this article. The remaining authors declare that the research was conducted in the absence of any commercial or financial relationships that could be construed as a potential conflict of interest.

## References

[1] WHO. Critically Important Antimicrobials for Human Medicine. Geneva: World Health Organization (2011).

[2] Ewers C Bethe A Semmler T Guenther S Wieler LH. Extended-spectrum beta-lactamase-producing and AmpC-producing *Escherichia coli* from livestock and companion animals, and their putative impact on public health: a global perspective. Clin Microbiol Infect. (2012) 18:646–55. 10.1111/j.1469-0691.2012.03850.x 22519858

[3] Gonzalez-Zorn B Escudero JA. Ecology of antimicrobial resistance: humans, animals, food and environment. Int Microbiol. (2012) 15:101–9. 10.2436/20.1501.01.163 23847814

[4] De Briyne N Atkinson J Pokludova L Borriello SP. Antibiotics used most commonly to treat animals in Europe. Vet Rec. (2014) 175:325. 10.1136/vr.102462 24899065 PMC4215272

[5] Haenni M Saras E Metayer V Medaille C Madec JY. High prevalence of *bla*_CTX−M−1_/IncI1/ST3 and *bla*_CMY−2_/IncI1/ST2 plasmids in healthy urban dogs in France. Antimicrob Agents Chemother. (2014) 58:5358–62. 10.1128/AAC.02545-14 24982072 PMC4135814

[6] Ovejero CM Escudero JA Thomas-Lopez D Hoefer A Moyano G Montero N. Highly tigecycline-resistant klebsiella pneumoniae sequence type 11 (ST11) and ST147 isolates from companion animals. Antimicrob Agents Chemother. (2017) 61:e02640-16. 10.1128/AAC.02640-16 28396550 PMC5444156

[7] Ljungquist O Ljungquist D Myrenas M Ryden C Finn M Bengtsson B. Evidence of household transfer of ESBL-/pAmpC-producing *Enterobacteriaceae* between humans and dogs - a pilot study. Infect Ecol Epidemiol. (2016) 6:31514. 10.3402/iee.v6.31514 27330043 PMC4916256

[8] Ewers C Grobbel M Stamm I Kopp PA Diehl I Semmler T. Emergence of human pandemic O25:H4-ST131 CTX-M-15 extended-spectrum-beta-lactamase-producing *Escherichia coli* among companion animals. J Antimicrob Chemother. (2010) 65:651–60. 10.1093/jac/dkq004 20118165

[9] Blake DP Hillman K Fenlon DR Low JC. Transfer of antibiotic resistance between commensal and pathogenic members of the *Enterobacteriaceae* under ileal conditions. J Appl Microbiol. (2003) 95:428–36. 10.1046/j.1365-2672.2003.01988.x 12911689

[10] Carattoli A. Plasmids and the spread of resistance. Int J Med Microbiol. (2013) 303:298–304. 10.1016/j.ijmm.2013.02.001 23499304

[11] Damborg P Gaustad IB Olsen JE Guardabassi L. Selection of CMY-2 producing *Escherichia coli* in the faecal flora of dogs treated with cephalexin. Vet Microbiol. (2011) 151:404–8. 10.1016/j.vetmic.2011.03.015 21497459

[12] Moreno A Bello H Guggiana D Dominguez M Gonzalez G. Extended-spectrum beta-lactamases belonging to CTX-M group produced by *Escherichia coli* strains isolated from companion animals treated with enrofloxacin. Vet Microbiol. (2008) 129:203–8. 10.1016/j.vetmic.2007.11.011 18166282

[13] Kimura A Yossapol M Shibata S Asai T. Selection of broad-spectrum cephalosporin-resistant *Escherichia coli* in the feces of healthy dogs after administration of first-generation cephalosporins. Microbiol Immunol. (2017) 61:34–41. 10.1111/1348-0421.12466 28111794

[14] Hordijk J Schoormans A Kwakernaak M Duim B Broens E Dierikx C. High prevalence of fecal carriage of extended spectrum beta-lactamase/AmpC-producing *Enterobacteriaceae* in cats and dogs. Front Microbiol. (2013) 4:242. 10.3389/fmicb.2013.00242 23966992 PMC3745002

[15] Damborg P Morsing MK Petersen T Bortolaia V Guardabassi L. CTX-M-1 and CTX-M-15-producing *Escherichia coli* in dog faeces from public gardens. Acta Vet Scand. (2015) 57:83. 10.1186/s13028-015-0174-3 26608707 PMC4660786

[16] Belas A Salazar AS Gama LT Couto N Pomba C. Risk factors for faecal colonisation with *Escherichia coli* producing extended-spectrum and plasmid-mediated AmpC beta-lactamases in dogs. Vet Rec. (2014) 175:202. 10.1136/vr.101978 24943100

[17] Hansen KH Bortolaia V Nielsen CA Nielsen JB Schonning K Agerso Y. Host-specific patterns of genetic diversity among IncI1-Iγ and IncK plasmids encoding CMY-2 beta-lactamase in *Escherichia coli* isolates from humans, poultry meat, poultry, and dogs in denmark. Appl Environ Microbiol. (2016) 82:4705–14. 10.1128/AEM.00495-16 27235431 PMC4984282

[18] Boehmer T Vogler AJ Thomas A Sauer S Hergenroether M Straubinger RK. Phenotypic characterization and whole genome analysis of extended-spectrum beta- lactamase-producing bacteria isolated from dogs in Germany. PLoS ONE. (2018) 13:e0206252. 10.1371/journal.pone.0206252 30365516 PMC6203360

[19] CLSI Performance Standards for Antimicobial Disk Susceptibility Tests. Approved Standard-Tenth Edition. Document M02-A10. Wayne, PA: CALS Institute (2009).

[20] CLSI Performance Standards for Antimicrobial Testing, 19th Informational Supplement. Document M100-S19. Wayne, PA: CALS Institute (2009).

[21] Derbyshire H Kay G Evans K Vaughan C Kavuri U Winstanley T. A simple disc diffusion method for detecting AmpC and extended-spectrum beta-lactamases in clinical isolates of *Enterobacteriaceae*. J Antimicrob Chemother. (2009) 63:497–501. 10.1093/jac/dkn535 19155228

[22] Dupouy V Doublet B Arpaillange N Praud K Bibbal D Brugere H. Dominant plasmids carrying extended-spectrum beta-lactamases blaCTX-M genes in genetically diverse *Escherichia coli* from slaughterhouse and urban wastewaters. Environ Microbiol Rep. (2016) 8:789–97. 10.1111/1758-2229.12440 27402421

[23] Dierikx CM Van Duijkeren E Schoormans AH Van Essen-Zandbergen A Veldman K Kant A. Occurrence and characteristics of extended-spectrum-beta-lactamase- and AmpC-producing clinical isolates derived from companion animals and horses. J Antimicrob Chemother. (2012) 67:1368–74. 10.1093/jac/dks049 22382469

[24] Kado CI Liu ST. Rapid procedure for detection and isolation of large and small plasmids. J Bacteriol. (1981) 145:1365–73. 7009583 10.1128/jb.145.3.1365-1373.1981 PMC217141

[25] Carattoli A Bertini A Villa L Falbo V Hopkins KL Threlfall EJ. Identification of plasmids by PCR-based replicon typing. J Microbiol Methods. (2005) 63:219–28. 10.1016/j.mimet.2005.03.018 15935499

[26] Schmidt VM Pinchbeck GL Nuttall T Mcewan N Dawson S Williams NJ. Antimicrobial resistance risk factors and characterisation of faecal *E. coli* isolated from healthy Labrador retrievers in the United Kingdom. Prev Vet Med. (2015) 119:31–40. 10.1016/j.prevetmed.2015.01.013 25732912

[27] Leite-Martins LR Mahu MI Costa AL Mendes A Lopes E Mendonca DM. Prevalence of antimicrobial resistance in enteric *Escherichia coli* from domestic pets and assessment of associated risk markers using a generalized linear mixed model. Prev Vet Med. (2014) 117:28–39. 10.1016/j.prevetmed.2014.09.008 25294317

[28] Espinosa-Gongora C Shah SQ Jessen LR Bortolaia V Langebaek R Bjornvad CR. Quantitative assessment of faecal shedding of beta-lactam-resistant *Escherichia coli* and enterococci in dogs. Vet Microbiol. (2015) 181:298–302. 10.1016/j.vetmic.2015.10.004 26494111

[29] Wedley AL Dawson S Maddox TW Coyne KP Pinchbeck GL Clegg P. Carriage of antimicrobial resistant *Escherichia coli* in dogs: prevalence, associated risk factors and molecular characteristics. Vet Microbiol. (2017) 199:23–30. 10.1016/j.vetmic.2016.11.017 28110781

[30] Lindemann PC Risberg K Wiker HG Mylvaganam H. Aminoglycoside resistance in clinical *Escherichia coli* and *Klebsiella pneumoniae* isolates from Western Norway. APMIS. (2012) 120:495–502. 10.1111/j.1600-0463.2011.02856.x 22583362

[31] Marti E Balcazar JL. Real-Time PCR assays for quantification of qnr genes in environmental water samples and chicken feces. Appl Environ Microbiol. (2013) 79:1743–5. 10.1128/AEM.03409-12 23275512 PMC3591933

[32] Zong Z Ginn AN Dobiasova H Iredell JR Partridge SR. Different IncI1 plasmids from *Escherichia coli* carry ISEcp1-blaCTX-M-15 associated with different Tn2-derived elements. Plasmid. (2015) 80:118–26. 10.1016/j.plasmid.2015.04.007 25929173

[33] Madec JY Haenni M Metayer V Saras E Nicolas-Chanoine MH High prevalence of the animal-associated *bla*_CTX−M−1_ IncI1/ST3 plasmid in human *Escherichia coli* isolates. Antimicrob Agents Chemother. (2015) 59:5860–1. 10.1128/AAC.00819-15 26124170 PMC4538533

[34] Zurfluh K Jakobi G Stephan R Hachler H Nuesch-Inderbinen M Replicon typing of plasmids carrying *bla*_CTX−M−1_ in *Enterobacteriaceae* of animal, environmental and human origin. Front Microbiol. (2014) 5:555 10.3389/fmicb.2014.00555 25400623 PMC4214192

[35] Jones-Dias D Manageiro V Martins AP Ferreira E Canica M. New class 2 Integron In2-4 among IncI1-positive *Escherichia coli* isolates carrying ESBL and PMAβ genes from food animals in Portugal. Foodborne Pathog Dis. (2016) 13:36–9. 10.1089/fpd.2015.1972 26575358

[36] Ben Sallem R Ben Slama K Rojo-Bezares B Porres-Osante N Jouini A Klibi N. IncI1 plasmids carrying bla(CTX-M-1) or bla(CMY-2) genes in *Escherichia coli* from healthy humans and animals in Tunisia. Microb Drug Resist. (2014) 20:495–500. 10.1089/mdr.2013.0224 24826863

[37] Madec JY Poirel L Saras E Gourguechon A Girlich D Nordmann P Non-ST131 *Escherichia coli* from cattle harbouring human-like *bla*_CTX−M−15_-carrying plasmids. J Antimicrob Chemother. (2012) 67:578–81. 10.1093/jac/dkr542 22210752

[38] Arvand M Bettge-Weller G Fruth A Uphoff H Pfeifer Y. Extended-spectrum beta-lactamase-producing Shiga toxin gene (stx1)-positive *Escherichia coli* O91:H14 carrying *bla*_CTX−M−15_ on an IncI1-ST31 plasmid isolated from a human patient in Germany. Int J Med Microbiol. (2015) 305:404–7. 10.1016/j.ijmm.2015.03.003 25801683

[39] Accogli M Fortini D Giufre M Graziani C Dolejska M Carattoli A. IncI1 plasmids associated with the spread of CMY-2, CTX-M-1 and SHV-12 in *Escherichia coli* of animal and human origin. Clin Microbiol Infect. (2013) 19:E238–40. 10.1111/1469-0691.12128 23331857

[40] Enterobase (2018). Available online at: http://enterobase.warwick.ac.uk/species/ecoli/search_strains (accessed March 26, 2019).

[41] Pietsch M Irrgang A Roschanski N Brenner Michael G Hamprecht A Rieber H. Whole genome analyses of CMY-2-producing *Escherichia coli* isolates from humans, animals and food in Germany. BMC Genomics. (2018) 19:601. 10.1186/s12864-018-4976-3 30092762 PMC6085623

[42] Liakopoulos A Mevius D Ceccarelli D. A review of SHV extended-spectrum beta-lactamases: neglected yet ubiquitous. Front Microbiol. (2016) 7:1374. 10.3389/fmicb.2016.01374 27656166 PMC5011133

[43] Alonso N Miro E Pascual V Rivera A Simo M Garcia MC. Molecular characterisation of acquired and overproduced chromosomal *bla*AmpC in *Escherichia coli* clinical isolates. Int J Antimicrob Agents. (2016) 47:62–8. 10.1016/j.ijantimicag.2015.10.007 26607336

[44] Dahmen S Haenni M Chatre P Madec JY. Characterization of *bla*_CTX−M_ IncFII plasmids and clones of *Escherichia coli* from pets in France. J Antimicrob Chemother. (2013) 68:2797–801. 10.1093/jac/dkt291 23852541

[45] Zogg AL Simmen S Zurfluh K Stephan R Schmitt SN Nuesch-Inderbinen M. High prevalence of extended-spectrum beta-lactamase producing *Enterobacteriaceae* among clinical isolates from cats and dogs admitted to a Veterinary Hospital in Switzerland. Front Vet Sci. (2018) 5:62. 10.3389/fvets.2018.00062 29662886 PMC5890143

[46] Blanco M Alonso MP Nicolas-Chanoine MH Dahbi G Mora A Blanco JE. Molecular epidemiology of *Escherichia coli* producing extended-spectrum {beta}-lactamases in Lugo (Spain): dissemination of clone O25b:H4-ST131 producing CTX-M-15. J Antimicrob Chemother. (2009) 63:1135–41. 10.1093/jac/dkp122 19351692

[47] Courpon-Claudinon A Lefort A Panhard X Clermont O Dornic Q Fantin B. Bacteraemia caused by third-generation cephalosporin-resistant *Escherichia coli* in France: prevalence, molecular epidemiology and clinical features. Clin Microbiol Infect. (2011) 17:557–65. 10.1111/j.1469-0691.2010.03298.x 20649802

[48] ECDC The European Union Summary Report on Antimicrobial Resistance in Zoonotic and Indicator Bacteria From Humans, Animals and Food in 2014. European Centre for Disease Prevention and Control (2016). 10.2903/j.efsa.2018.5182 PMC7009656 32625816

[49] Hidalgo L Gutierrez B Ovejero CM Carrilero L Matrat S Saba CK. *Klebsiella pneumoniae* sequence type 11 from companion animals bearing ArmA methyltransferase, DHA-1 beta-lactamase, and QnrB4. Antimicrob Agents Chemother. (2013) 57:4532–4. 10.1128/AAC.00491-13 23752506 PMC3754351

